# Recurring Cystic Tumors of the Supra-Scapular Region

**Published:** 1883-04

**Authors:** J. Lindsay

**Affiliations:** Huntington, L. I.


### VI.—RECURRING CYSTIC TUMORS OF THE SUPRA-SCAPULAR REGION.

BY J. LINDSAY, D.V.S.

On August 31st a horse was driven to my office from Northport, L. I. He was suffering from two very large cystic tumors, one on each side, just over the superior border of the scapula. The two were so large that they met at the dorsal line, thus forming a double tumor which projected upwards very much like a hump upon a camel's back.

I opened them and they discharged a liquid of a yellow color, and somewhat resembled synovial fluid, and contained flakes resembling those seen in curdy pus. The total amount of the fluid was about three quarts.

After their contents had escaped I injected a solution of zinci sulphate (ʒv. to ℥ii.) into the sac of each tumor, and then plugged the wound with oakum.

As I could not see the animal frequently I told the owner how to inject the cavity twice daily, and keep them plugged with fresh oakum, I also ordered rest and when put to work to use a Dutch instead of a Hame Collar, as I was inclined to think the ill fitting collar the cause of the trouble.

At this time the horse was much reduced in flesh, had been worked hard, but otherwise appeared to be well and strong. He had been fed far more than his mate, yet he was thin and his mate in good condition. All this time, however he had a ferocious appetite yet failed. It was thought from the appetite that he might have intestinal worms.

On account of the impoverished condition and the ichorous discharge from the tumors, I gave him ʒi of Ferri Sulphate with his food twice daily.

From this time until Dec. 31st I never saw or heard from the horse, but on this day, he was again brought to me for treatment for another cystic tumor, located upon the left shoulder at the middle of the scapula.

This growth seemed to be of the same nature as the former neoplasms, aud when opened discharged the same kind of fluid. At this time his general condition was remarkably good, he was fat and slick, and his nostrils looked bright and normally red. The former wounds had healed with very slight scars. The old tumors had evidently perfectly healed, and the present one originated wholly independent of the first.

January 20th, was called to see the horse, for after the healing of the third a fourth appeared at the summit of the withers and the owner opened it, but it failed to heal. When I came to examine, a small sinus was noticed, from which there was a slight and thin discharge. Upon opening this sinus it was found that the scapular portion had been undermined and at the bottom there was quite a large sac.

I opened this sac by a long incision from the superior opening to the bottom of the anterior border of the scapula and thus exposed the whole cavity, and ordered it washed daily with a solution of Carbolic Acid and Glycerine.

The wound was coverd with a piece of muslin to keep out dirt. On the 26th the wound was so nearly healed that I left him to the care of the owner.

February 21st, passing by, I stopped in to see him, and found the wound almost healed and the horse in the best of condition and spirits.

On the 24th the owner called to say that the swelling was returning with a rising on the back of the neck, and wished me to call.

As there seemed to be no pressing necessity, I did not see the horse until the 26th, and found him in this condition. Breathing very labored, weak and shallow. Pulse hardly perceptible. Temperature, 105° F. Animal very weak. Swelling hard, extending from the neck down both shoulders and side of back as far as the hips. The swelling which radiated from the common centre had a doughy odematous feel.

As there appeared to be no chance to save the animal I ordered him destroyed. He was led about 300 yards from the stable for this purpose.

Owing to his extreme weakness it was almost impossible to get him there, and it was necessary to stop and rest several times on the way.

Owing to the severity of the weather no autopsy was held.

HUNTINGTON, L. I.

[As the character of the cyst walls was not described, nor the immediate surrounding tissue, it is absolutely impossible for us to state positively the nature of the growth. It seems quite probable, however, that they were malignant in their nature on account of frequency with which they recurred. The writer speaks of having seen similar cases and states that they always occur in weak and debilitated animals. This fact would suggest a scrofulous or streuous nature. The cause of death, we should be inclined to think, was due to a diffuse cellulitis septicæmia, and possibly pyæmia. A more careful study of such cases will have to be made to clearly elucidate the pathology of such conditions. Another interesting fact in connection with this case is that when the owner tried his hand at surgical treatment it was not so successful as in the hands of a scientific veterinarian. This fact ought to be encouragement of the positive kind to the student and practitioner, and teach the laity that a good veterinarian is well worth employing at all times—ED.]

